# Utility of endoscopic anatomical optical coherence tomography in functional rhinoplasty

**DOI:** 10.1117/1.JBO.25.1.016001

**Published:** 2020-01-07

**Authors:** Santosh Balakrishnan, Ruofei Bu, Candace M. Waters, Bryan M. Brandon, Julia S. Kimbell, Wesley H. Stepp, William W. Shockley, J. Madison Clark, Amy L. Oldenburg

**Affiliations:** a University of North Carolina at Chapel Hill, Department of Biomedical Engineering, Chapel Hill, North Carolina, United States; b University of North Carolina School of Medicine, Department of Otolaryngology/Head and Neck Surgery, Chapel Hill, North Carolina, United States; c University of North Carolina at Chapel Hill, Department of Physics and Astronomy, Chapel Hill, North Carolina, United States; d University of North Carolina at Chapel Hill, Biomedical Research Imaging Center, Chapel Hill, North Carolina, United States

**Keywords:** optical coherence tomography, endoscopic OCT, nasal valve compromise, functional rhinoplasty, nasal valve surgery

## Abstract

Objective measurement of the nasal valve region is valuable for the assessment of functional rhinoplasty surgical outcomes. Anatomical optical coherence tomography (aOCT) is an imaging modality that may be used to obtain real-time, quantitative, and volumetric scans of the nasal airway. We aim to evaluate if volumetric aOCT imaging is useful for the examination of the nasal valve region before and after functional rhinoplasty procedures. aOCT scans of the nasal valves were performed on four cadaveric heads before and after spreader graft and butterfly graft procedures. The resulting aOCT images were compared against video endoscopy images, and the segmented volumes of the nasal airway obtained from aOCT scans were compared with computed tomography (CT) derived volumes acquired under the same conditions. The aOCT-derived volumes match the CT volumes closely, with a mean Dice similarity coefficient of 0.88 and a mean Hausdorff distance of 2.3 mm. Furthermore, the aOCT images were found to represent the shape of the nasal cavity accurately. Due to its ability to perform real-time, quantitative, and accurate evaluation of the nasal airway, aOCT imaging is a promising modality for the objective assessment of the nasal valves before and after functional rhinoplasty procedures.

## 1 Introduction

Nasal airway obstruction impairs airflow through the nose and is a common otorhinolaryngologic complaint.^1^ Nasal valve compromise (NVC) is an anatomical cause of nasal obstruction and the primary target for most functional rhinoplasty procedures.^2^ NVC is diagnosed, and treatment outcomes are evaluated using a combination of physical examination and patient-reported subjective measures.^3^ While physical examination findings and patient-reported outcome measures are mainstay indicators of a successful intervention,^3^ it is often preferable to obtain quantitative, objective assessments. Such precision is particularly useful for surgical planning, for assessing the outcomes of surgical interventions, for understanding of complex cases, and for research studies.^1^^,^^4^

Methods for obtaining anatomical, objective measures of nasal obstruction include acoustic rhinometry (AR) and imaging studies with computed tomography (CT) or magnetic resonance imaging (MRI). AR can be used to determine the minimum cross-sectional area (CSA) and the volume of the nasal passage, whereas CT and MRI determine the structure, shape, and volume of the nasal airway.^5^ However, these methods only yield a static measurement of nasal dimensions. They cannot measure the dynamic deformation of the airway during breathing nor take into account factors such as nasal congestion.^5^ Physiologic measures such as nasal airway resistance or peak inspiratory flow only yield a global assessment of nasal patency.^5^ Anatomically accurate, three-dimensional (3-D) computational models generated from high-resolution imaging are needed to perform computational fluid dynamics (CFD) simulations of nasal airflow. CFD simulations can yield localized physiological measures, such as flow rate, resistance, heat transfer, and air humidification. However, such simulations are usually based on 3-D models derived from fine-cut CT scans and thus involve subjecting patients to unnecessary radiation exposure.^2^

Optical coherence tomography (OCT) is an imaging modality that provides cross-sectional imaging of biological tissues by measuring the magnitude and echo time delay of backscattered light using low-coherence interferometry.^6^ Long-range or anatomical OCT (aOCT) is a technological variant of OCT with a fiber optic imaging probe that can be deployed endoscopically and offers longer imaging distances than conventional OCT.^7^ Englhard et al.^8^^,^^9^ have shown that long-range OCT may be used to quantify the internal nasal valve (INV) geometry and to study the dynamic behavior of the valve during respiration. The hypothesis of this study was that volumetric aOCT imaging is an accurate modality for the quantitative anatomical assessment of the nasal valve region for functional rhinoplasty procedures. The nasal valve region is a complex anatomical structure,^10^ and a technology that allows for real-time, quantitative, volumetric assessment of the nasal airway would complement existing measures and allow for better diagnosis, surgical therapy planning, and assessment of postsurgical outcomes.

Functional rhinoplasty procedures for the treatment of NVC are performed to increase the CSA of the nasal valve or to reinforce the lateral wall to prevent collapse. Examples of such nasal valve repair procedures include batten grafts, spreader grafts (SG), butterfly grafts (BFG), and suture suspension.^11^ Measurements such as rhinomanometry, AR, and peak inspiratory flow have been used to report the outcomes of such nasal valve repair surgeries objectively.^11^ This paper describes an experiment in which aOCT imaging was performed before and after SG and BFG procedures in cadaveric models to obtain anatomical, objective measurements of the nasal airway. In order to evaluate the accuracy of nasal airway volume and INV shape obtained from aOCT scans, comparisons were made with CT-derived volumes and to nasal video endoscopic frames. Our findings highlight the potential of aOCT imaging for rapid and accurate assessment of the nasal airway in functional rhinoplasty procedures.

## 2 Methods

**Fig. 1:**
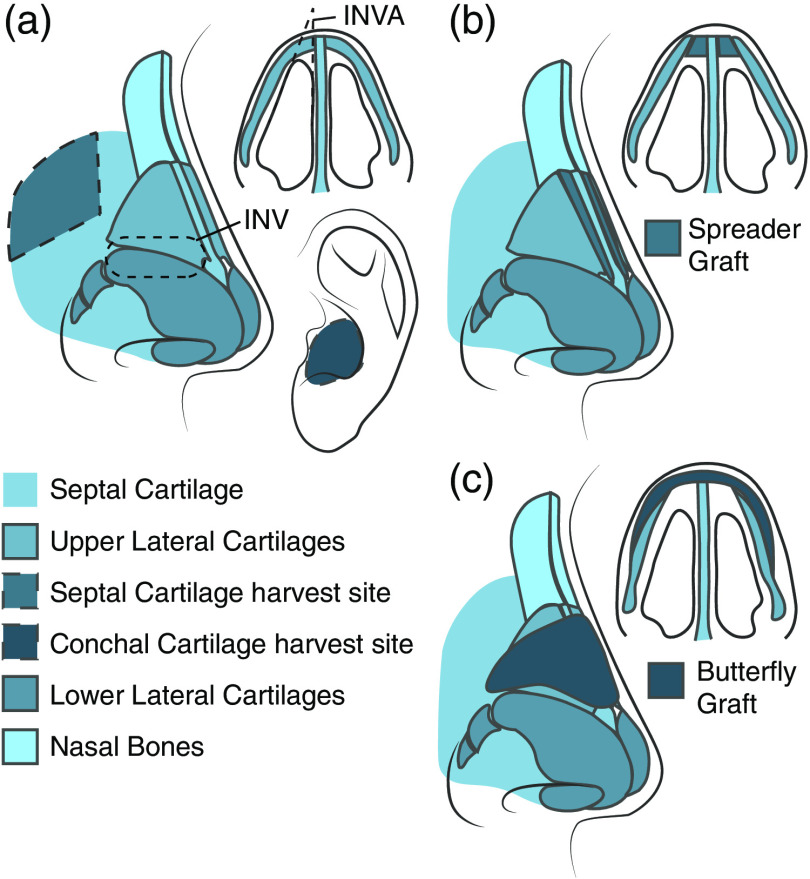
Overview of the nasal anatomy and surgical procedures. (a) Anatomy of the nose depicting the major cartilages, the sites of the cartilage harvest, and the nasal bones. INV, internal nasal valve; INVA, angle of the INV. (b) Placement of the grafts between the ULC and the septum for the spreader graft procedure and (c) placement of the onlay graft over the ULC for the butterfly graft procedure.

SG and BFG are two common functional rhinoplasty procedures that focus on widening the nasal valve to treat NVC.^11^ In a bilateral SG procedure, a segment of the nasal septal cartilage (SC) is first harvested and shaped into two rectangular cartilage grafts. These grafts are then placed and sutured between the upper lateral cartilages (ULC) and the septum; the grafts widen the CSA of the valve by lateralizing the insertion of the ULC.^12^ In a BFG procedure, a concave, pyramid-shaped graft is first fabricated from a harvested piece of conchal cartilage. This graft is placed over the nasal septum and sutured to the caudal margins of the ULC such that the natural spring of the graft helps both to widen the nasal valve and to reinforce the strength of the ULC.^13^ Figure 1 shows the nasal anatomy and gives an overview of the cartilage harvest and graft placements in SG and BFG procedures.

For this study, four adult cadaveric heads (caucasian; 2 male, 2 female; aged between 65 and 86 years) were obtained (Science Care Inc., Arizona), and the nasal cavities were cleansed to remove any debris. Baseline scans using both CT and aOCT were performed on each head prior to functional rhinoplasty procedures. Septoplasty was performed prior to the baseline scans, if clinically indicated by a physical exam and for the harvest of spreader grafts. Bilateral SG and BFG procedures, as described above, were performed in alternating order on each donor head. SCs were harvested from donors 1 and 3, and conchal cartilages were harvested from donors 2 and 4; these harvested cartilages were used to form grafts that were subsequently used for the SG and BFG procedures on all heads. After each procedure, the soft-tissue envelope was redraped, all incisions were closed and CT, aOCT scans were repeated. The institutional review board at the University of North Carolina School of Medicine, Chapel Hill, determined that this study was exempt from review due to the use of only cadaveric tissue.

**Fig. 2:**
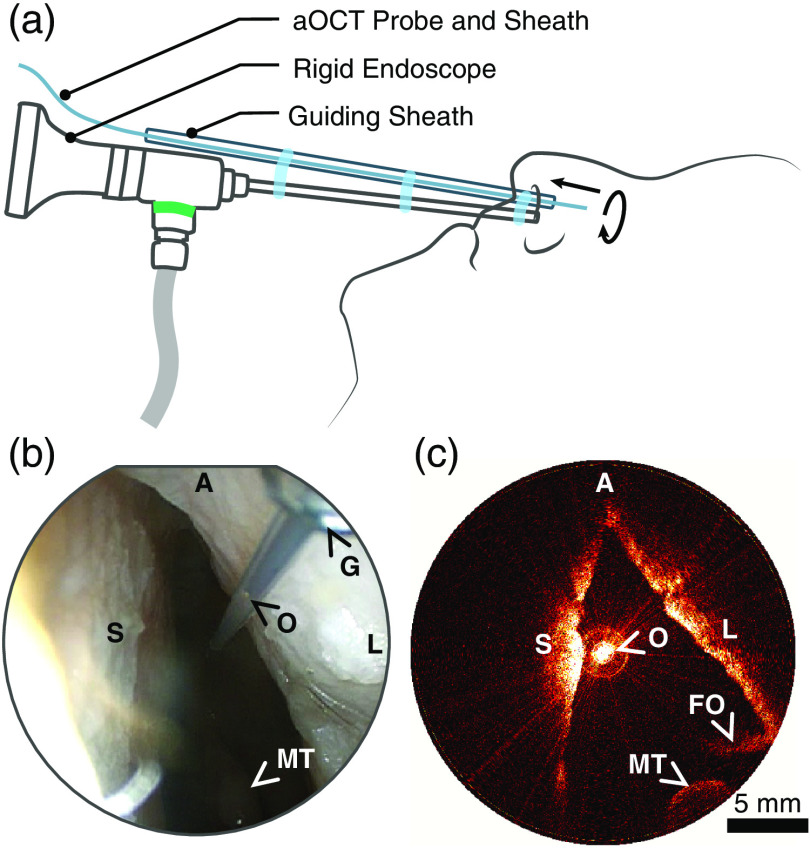
(a) Setup used to perform aOCT imaging of the nasal airways; (b) nasal endoscopy image depicting the positioning of the aOCT probe and sheaths in the left nasal cavity, preintervention. O, tip of the aOCT probe seen within the protective sheath at the conclusion of a pullback scan; G, tip of the guiding sheath; MT, middle turbinate; A, anterior; S, septum; L, lateral wall. (c) Representative aOCT image acquired in the left nasal cavity of the same patient, preintervention. O, aOCT probe tip/origin of the acquired aOCT image; MT, middle turbinate; FO, fold-over/aliasing artifact from nasal structures beyond the aOCT system’s maximum imaging radius of 12 mm. A, anterior; S, septum; and L, lateral wall.

CT scans were performed with a 3-D Accuitomo F170 cone beam CT system (J. Morita Mfg. Corp., Japan) with an isotropic voxel size of 0.33 mm. The aOCT scans were performed with a cart-based swept source aOCT system with a center wavelength of 1300 nm, A-line rate of 100 kHz, axial resolution of ∼13  μm, peak sensitivity of ∼106  dB, and an imaging range of 12 mm.^14^ The sample arm consisted of an endoscopic, fiber-optic probe ∼143  cm in length with a side-looking beam. The probe, encased in a Nitinol tube driveshaft, was placed in a sealed protective sheath; the final outer diameter of the aOCT probe assembly was ∼0.86  mm. The power at the output of the probe was ∼13  mW. Helical scans were performed by rotating and translating the aOCT probe within the stationary protective sheath. In order to perform scans of the nasal airway, a stiff guiding sheath was attached to a 0-deg straight, nasal endoscope (diameter 4 mm, length 18 cm; Karl Storz SE & Co., Germany). The aOCT catheter was introduced into the nasal airway through the guiding sheath and positioned within the nasal airway under a combination of video endoscopic guidance and real-time aOCT imaging (Fig. 2). aOCT volumetric scans of the cartilaginous vault of the nasal cavity were performed over a pullback distance of ∼20  mm by rotating and translating the aOCT probe at 20 Hz and 6 mm/s respectively, resulting in an image stack of 67 frames with a pitch of 0.3 mm. aOCT scans were performed five times on each head before and after each of the two interventions, separately on the left and right nasal airway to yield 120 scans in total during this study.

**Fig. 3:**
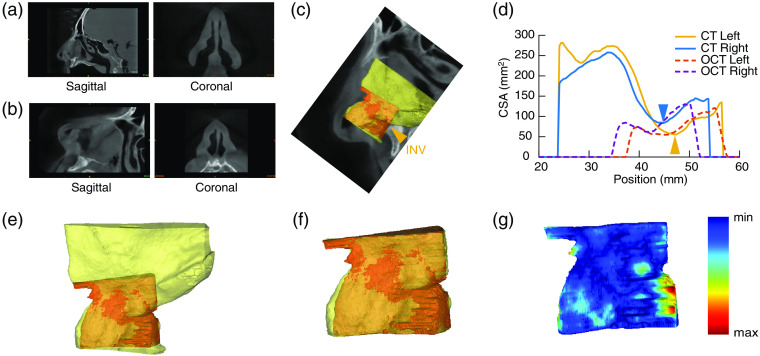
Data processing steps and representative results. (a) CT sagittal and coronal views in the traditional plane of acquisition; (b) CT sagittal and coronal views after resampling in the plane perpendicular to the airflow direction. (c) Registered CT (yellow color) and aOCT (orange color) volumes of the left nasal airway overlaid on the sagittal image for visualization along with the estimate of the left INV plane from (d). (d) CSA of the airway in the direction perpendicular to the airflow as determined from the segmented CT and aOCT volumes; the position with the minimum CSA on the CT scans was selected as an estimate of the INV (arrowheads). (e) Registered, overlaid CT (yellow color), and aOCT (orange color) volumes; (f) overlaid CT and aOCT volumes with the CT volume modified to account for the aOCT scan’s field of view. (g) Hausdorff distances between the two volumes in (f) shown on the aOCT volume using a colormap.

Traditional CT scans acquired and reconstructed in a plane perpendicular to the hard palate [Fig. 3(a)] are not appropriate for the assessment of the nasal valve;^15^^–^^18^ therefore, the acquired CT scans were first resampled in a plane perpendicular to the airflow direction [Fig. 3(b)] using a method similar to that described by Bloom et al.^15^ and Suh et al.^16^ The resampled dataset had isotropic voxels 0.33 mm in size. The nasal airway was segmented by thresholding and the segmented CT slices were used to generate a 3-D volume of the nasal airway [Fig. 3(e), yellow color]. The repeated aOCT scans acquired on each head before and after each of the two interventions were qualitatively assessed for reproducibility, and one scan on each side was chosen for further processing; the chosen scan was segmented, and the segmentation masks were used to produce the aOCT-derived 3-D volume of the airway [Fig. 3(e), orange color]. The aOCT and CT volumes were registered by minimization of the least square distance to obtain volumes of the airway from both modalities on a common coordinate system [Figs. 3(c) and 3(e)]. The INV is the narrowest segment of the nasal airway with the smallest CSA;^1^ the segmented CT nasal airways were analyzed and the slice with the minimum CSA, in the plane perpendicular to the airflow direction on each side, was considered to be the best estimate of the INV for both the CT and aOCT volumes. All of the image processing steps described here were performed using Mimics Research 18.0 (Materialise NV, Belgium).

In order to assess the accuracy of the aOCT imaging, several qualitative and quantitative assessments were performed. The overall shape of the nasal cavity as seen on nasal video endoscopy was compared to that obtained by the aOCT scans. The aOCT volumes, after registration with the CT volumes, were overlaid and visually assessed for similarity [Fig. 3(e)]. The registered aOCT volumes were also used to obtain contours of the nasal airway in the plane perpendicular to the airflow direction, and these were compared to the resampled CT coronal images at the level of the INV. In order to perform quantitative comparisons between the CT and aOCT 3-D volumes, it was required to modify the CT volume to account for the aOCT acquisition’s limited field of view. The aOCT system’s maximum imaging radius was 12 mm, and since aOCT images are produced with the probe tip at the origin, the volume of the nasal airway captured on the aOCT scans depends on the position of the aOCT probe within the airway. In order to account for this limitation as well as for the pullback length of the aOCT scans, the corresponding CT volumes were reduced to match the aOCT field of view [Fig. 3(f)] before performing quantitative comparisons. Specifically, CT volumes were clipped in the posterior, cranial and caudal directions based on the extent of the registered aOCT volumes; no modifications were made in other three directions. The Dice similarity coefficient (DSC) was used to quantify the overall segmented volume similarity between the two modalities and the agreement between the segmentation contours of each set of paired volumes was assessed using the Hausdorff distance (HD) [Fig. 3(g)].^19^

## 3 Results

**Fig. 4:**
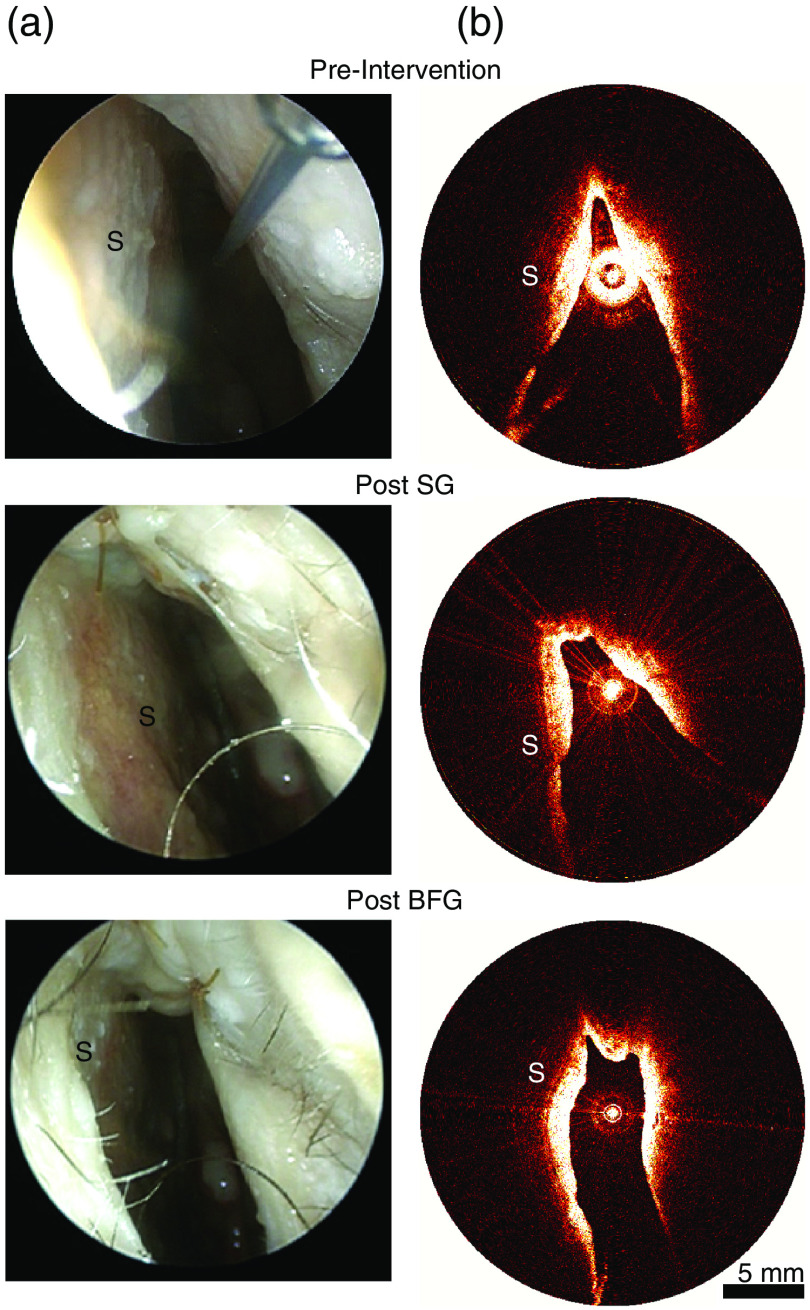
Representative (a) endoscopic and (b) aOCT images acquired before and after the interventions in the left nasal cavity. aOCT images demonstrate similar morphology of the INV region, including widening of the valve apex after surgical intervention. SG, spreader graft; BFG, butterfly graft; S, septum.

**Fig. 5:**
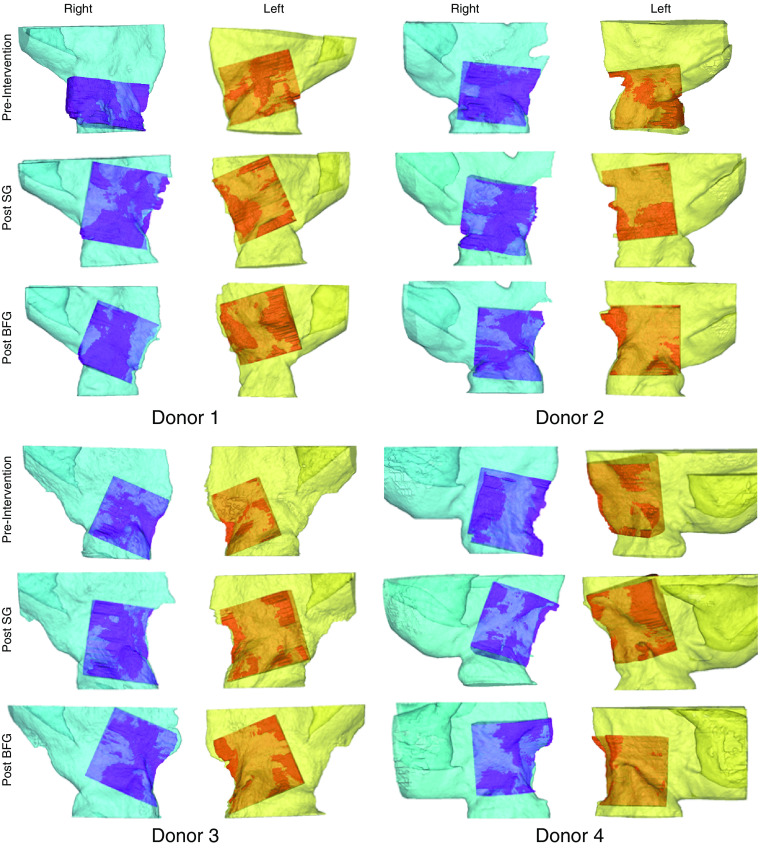
Comparison of the nasal airway volumes derived from CT (lighter color) and aOCT (darker color) scans. SG, spreader graft; BFG, butterfly graft.

The shape of the nasal cavity on aOCT images and on the video nasal endoscopy frames was visually similar (Fig. 4) for all heads before and after each intervention. The comparison of the aOCT and CT volumes also yielded promising results. In general, the registered, overlaid volumes demonstrate a high degree of similarity (Fig. 5) for the region of the nasal airway in the aOCT system’s field of view.

The quantitative comparison of the aOCT and the modified CT volumes showed that the difference in volumes across all measurements was $48.9\pm 145.7\;{\mathrm{mm}}^{3}$ (all results reported as mean ± standard deviation) or 2.2±9.4% of the CT volume, the DSC between the compared volumes was 0.88±0.04, and the HD was 2.3±0.6  mm.

**Fig. 6:**
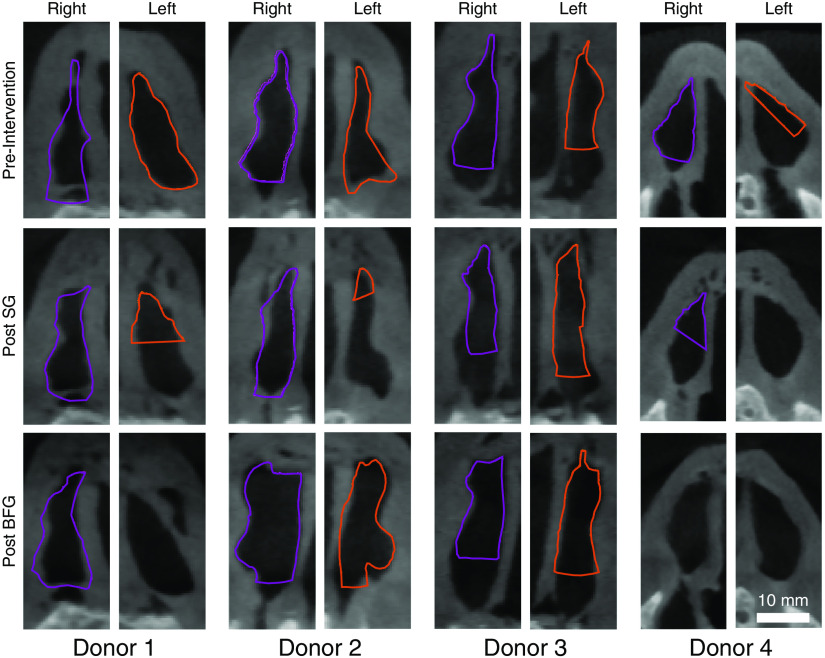
Contours of the INV determined from aOCT scans (purple, right INV; orange, left INV) overlaid on corresponding CT coronal images. SG, spreader graft; BFG, butterfly graft.

In the cases where the aOCT scan included the region of the INV, the contours of the INV (in the plane perpendicular to the airflow direction) derived from the registered aOCT volumes were found to correspond closely to the underlying nasal valve shape as determined from the CT scans (Fig. 6). The similarity in the INV angle between the aOCT contours and CT images was not quantified as the INV angle measured on the aOCT scans is expected to be identical to that on the CT scans due to the degree of agreement of the contours with the underlying nasal valve shape.

## 4 Discussion

In order for aOCT to be adopted for the assessment of the nasal valve region, either for the diagnosis of NVC or for the outcome assessment of functional rhinoplasty procedures, the imaging protocol has to be quick and easy to perform, and the results obtained have to be accurate. The imaging setup [Fig. 2(a)] utilized in this study allowed the physician to rapidly position the aOCT probe within the nasal airway as they had both the endoscopic and the aOCT image for guidance. In spite of these cues, the region of INV was missed on some scans: either partially (for example, on donor 1, post SG, left; see Fig. 6) or completely (for example, on donor 4, post BFG, left and right; see Fig. 6). We anticipate that with greater operator experience, there would be increased familiarity with the appearance of anatomical landmarks on the aOCT images. This in turn would allow the protocol for probe positioning and the aOCT scan pullback length to be optimally chosen such that the INV is consistently imaged. Each pullback, helical aOCT scan took ∼3  s; the total time taken for positioning each head and acquisition of the 10 aOCT scans on each head, either before or after the interventions, was 16±13  min. In scans that took longer to complete, the aOCT catheter had to be removed and cleaned to remove deposits that had accumulated on the protective sheath. We expect that it should be possible to improve the scan times in the future with better suctioning and positioning during the scans. The repeated aOCT scans were often performed without removing or repositioning of the aOCT probe and by the same operator; therefore, the datasets acquired during this study were not appropriate for the assessment of repeatability or reproducibility of our imaging protocol.

The limited imaging range of the aOCT system, the scan length, and the positioning of the probe tip within the cavity caused portions of the nasal airway to be missed on all scans (see Fig. 5). Due to this limitation, the CT volumes had to be modified to match the aOCT scan volumes as described in the methods section [see Figs. 3(e) and 3(f)]. Qualitative and quantitative comparisons shown in Sec. 3 (Fig. 5 and Table 1) demonstrate that the aOCT volumes faithfully depict the morphology of the nasal cavity and have the correct dimensions when compared to the modified CT volumes. While the volumes are similar between the two modalities, they are not exactly identical; the observed discrepancies could be explained by a number of factors. First, any differences in the soft tissue envelope position between the two scans would manifest as a difference in the volumes acquired by the two modalities. While the specimens were handled carefully during the study, it is possible that in some cases the soft tissue envelope was displaced slightly while transporting or positioning the heads during the CT and aOCT scans. In addition, segmentation of airway in the aOCT images was sometimes difficult as portions of the nasal walls were not always perfectly discernable. This was caused either by lower reflectivity of certain surfaces, caused either by a weak signal from surfaces at long radial distances due to sensitivity roll-off of the aOCT system^14^ or by shadowing from other structures of the nasal wall blocking the aOCT line-of-sight. Any errors in the aOCT segmentation would result in the aOCT volume not matching the CT volume perfectly in these affected regions. Finally, nonuniform rotational distortion (NURD) is a distortion that affects endoscopic probe-based OCT systems.^20^^,^^21^ While the effect of NURD on this experiment was negligible in most cases as the trajectory of aOCT probe had no tight curves, we suspect that volumes for donor 4, post BFG, left side showed poor agreement as compared to the other volume sets due to NURD. After excluding this datapoint, the difference in volume, DSC, and HD improve to $28.1\pm 106.6\;{\mathrm{mm}}^{3}$ or 1.1±7.6% of the CT volume, 0.89±0.04 and 2.2±0.5  mm, respectively.

**Table 1:** Summary of quantitative comparisons between the aOCT and the modified CT volumes. DSC, Dice similarity coefficient; HD, Hausdorff distance; SG, spreader graft; BFG, butterfly graft.

			Volume (${\mathrm{mm}}^{3}$)		DSC	HD (mm)
			CT	aOCT		
Donor 1	Left	Preintervention	1664.96	1699.89	0.905	2.087
		Post SG	1346.63	1345.12	0.872	2.721
		Post BFG	2464.13	2619.59	0.919	2.877
	Right	Preintervention	812.43	1002.79	0.790	2.721
		Post SG	1392.31	1343.94	0.873	2.113
		Post BFG	1446.61	1510.32	0.870	1.952
Donor 2	Left	Preintervention	1422.49	1481.11	0.872	2.310
		Post SG	1439.42	1467.60	0.788	2.701
		Post BFG	1829.23	1615.48	0.877	2.007
	Right	Preintervention	1360.61	1466.45	0.885	2.310
		Post SG	1558.48	1598.19	0.880	1.980
		Post BFG	2071.37	1894.49	0.928	1.896
Donor 3	Left	Preintervention	1117.53	1044.76	0.855	2.139
		Post SG	1879.07	1801.59	0.884	2.661
		Post BFG	1638.22	1639.52	0.883	3.006
	Right	Preintervention	1707.01	1523.98	0.898	1.320
		Post SG	1542.88	1493.47	0.917	1.896
		Post BFG	1446.79	1337.65	0.917	1.476
Donor 4	Left	Preintervention	1975.71	1925.79	0.885	2.970
		Post SG	2476.56	2486.88	0.930	1.867
		Post BFG	1829.80	1303.43	0.806	4.290
	Right	Preintervention	1875.48	1753.19	0.905	1.867
		Post SG	1815.61	1739.60	0.939	1.896
		Post BFG	1804.94	1650.41	0.911	1.896

Since the INV is the narrowest portion of the nasal airway, it was possible for the aOCT system to image this region completely when the probe was adequately centered (for example, donor 1, preintervention, left and right; see Fig. 6) even with its limited imaging range of 12 mm. However, in cases where the probe was not positioned centrally enough, the posterior portion of the INV was missed and the aOCT contours appear clipped in the posterior direction (for example, donor 3, post BFG, left and right; see Fig. 6). As a result, it was not possible to assess the improvement in CSA of the INV or the volume of the INV region due to the SG or BFG procedures. Since the apex of the nasal airway was always visible on the aOCT scans, it would have been possible to quantify treatment outcomes on the basis of the change in the INV angle.^8^^,^^9^ However, the measurement of a single INV angle for the complex nasal valve shapes seen in this study (Fig. 6) is unlikely to be robust or accurate.^10^^,^^22^ A longer aOCT imaging range would be desirable to allow for both the external and internal nasal valve regions to be scanned regardless of the positioning of the probe and to avoid the aliasing artifact shown in Fig. 2(c). This would allow for direct comparisons with CT without the need to clip the CT volumes in the posterior direction.

**Fig. 7:**
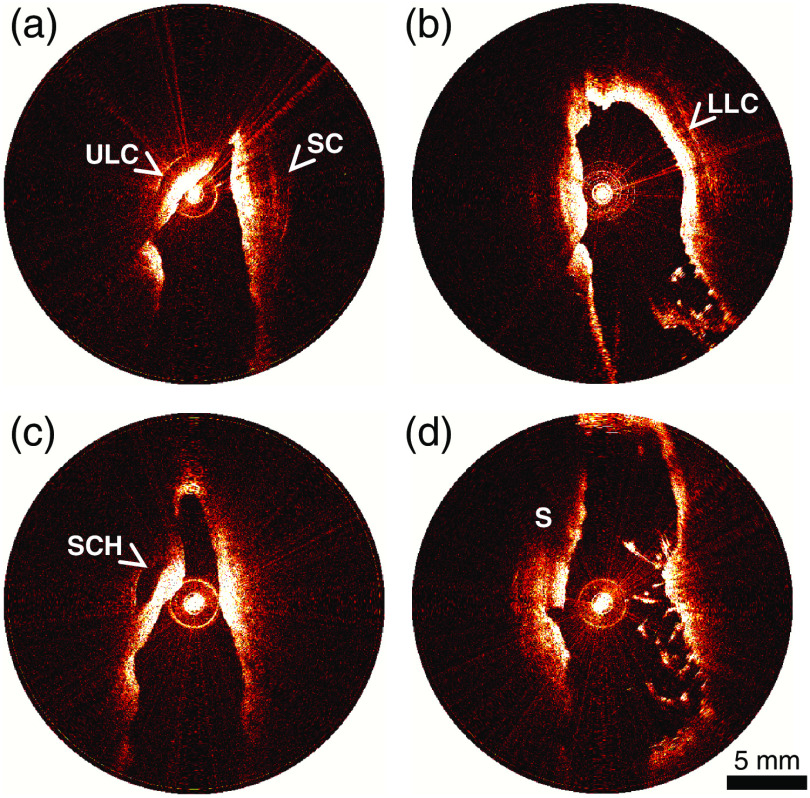
Representative aOCT images of the nasal airway: (a) the ULC and the SC are seen on this image of the right nasal cavity; (b) the LLC can be visualized in this image of the left nasal airway; (c) image acquired past the INV in the left nasal airway shows the site of the septal cartilage harvest (SCH); and (d) image acquired close to the level of the left external nasal valve. S, septum.

Despite the limitations noted above, we found aOCT imaging to be a valuable modality for the quantitative, objective assessment of the nasal valve region in this study; aOCT imaging was also found to have several unique advantages over the existing methods for the assessment of nasal patency. Volumetric assessment of the nasal valves may be performed with aOCT along with other tests in the physician’s office without having to schedule a separate exam or exposure to ionizing radiation as in the case of CT studies. Volumetric assessments of the nasal cavity are preferable since CSAs alone cannot predict the nature of the airflow dynamics, which could be turbulent, laminar, or vortex-like depending on the flow rate and nasal anatomy along the airflow path. The volumes generated from aOCT scans may therefore be subsequently used for performing CFD simulations with a goal of obtaining a more physiological assessment of nasal patency.^2^^,^^4^ In comparison with AR, aOCT imaging allows for the complete 3-D assessment of the nasal valves as opposed to a series of CSA measurements. In addition, aOCT is not affected by some of the known limitations of AR due to its use of reflection of acoustic waves;^18^ aOCT can image structures distal from the nostrils and can measure regions beyond narrow apertures without loss of accuracy. In contrast to nasal endoscopy, aOCT can produce cross-sectional images that are quantitative and not affected to the distortions commonly encountered in video endoscopy.^16^ aOCT scans are also performed in real-time, allowing for the assessment of static or dynamic obstruction of the nasal airway with respiration.^9^ Another distinctive feature of OCT imaging is its ability to see beneath the tissue surface up to a depth of ∼2  mm;^6^ during our study, this allowed us to visualize the upper lateral, lower lateral, and septal cartilages as well as the site of the SC harvest (see Fig. 7).

Based on the close agreement between the aOCT and CT volumes we observed during this study, we conclude that aOCT imaging is a valuable tool for the assessment of the nasal airway. The results obtained, as well as the unique advantages outlined above, make aOCT imaging a promising modality to explore for the diagnosis of NVC and for the assessment of functional rhinoplasty procedures. Additional studies are needed to further validate the accuracy of aOCT imaging of the nasal airway, to optimize the study protocol and explore other potential applications of aOCT imaging of the nasal valves.
